# Ribosome-associated Asc1/RACK1 is required for endonucleolytic cleavage induced by stalled ribosome at the 3′ end of nonstop mRNA

**DOI:** 10.1038/srep28234

**Published:** 2016-06-17

**Authors:** Ken Ikeuchi, Toshifumi Inada

**Affiliations:** 1 Graduate School of Pharmaceutical Science, Tohoku University, Aoba-ku, Sendai 980-8578, Japan

## Abstract

Dom34-Hbs1 stimulates degradation of aberrant mRNAs lacking termination codons by dissociating ribosomes stalled at the 3′ ends, and plays crucial roles in Nonstop Decay (NSD) and No-Go Decay (NGD). In the *dom34*Δ mutant, nonstop mRNA is degraded by sequential endonucleolytic cleavages induced by a stalled ribosome at the 3′ end. Here, we report that ribosome-associated Asc1/RACK1 is required for the endonucleolytic cleavage of nonstop mRNA by stalled ribosome at the 3′ end of mRNA in *dom34*Δ mutant cells. Asc1/RACK1 facilitates degradation of truncated *GFP-Rz* mRNA in the absence of Dom34 and exosome-dependent decay. Asc1/RACK1 is required for the sequential endonucleolytic cleavages by the stalled ribosome in the *dom34*Δ mutant, depending on its ribosome-binding activity. The levels of peptidyl-tRNA derived from nonstop mRNA were elevated in *dom34*Δ*asc1*Δ mutant cells, and overproduction of nonstop mRNA inhibited growth of mutant cells. E3 ubiquitin ligase Ltn1 degrades the arrest products from truncated *GFP-Rz* mRNA in *dom34*Δ and *dom34*Δ*asc1*Δ mutant cells, and Asc1/RACK1 represses the levels of substrates for Ltn1-dependent degradation. These indicate that ribosome-associated Asc1/RACK1 facilitates endonucleolytic cleavage of nonstop mRNA by stalled ribosomes and represses the levels of aberrant products even in the absence of Dom34. We propose that Asc1/RACK1 acts as a fail-safe in quality control for nonstop mRNA.

Systems for quality control of aberrant mRNAs prevent production of potentially harmful protein products by repressing translation and promoting protein degradation and stimulating mRNA degradation^1^,^2^,^3^,^4^,^5^. No-Go Decay (NGD) of mRNA, which is triggered by blockage of translation during the elongation step, induces the endonucleolytic cleavage in the vicinity of the stalled site^6^,^7^,^8^,^9^,^10^. This cleavage results in the production of two mRNA fragments, a 5′-NGD intermediate and a 3′-NGD intermediate, which are further degraded by the exosome and Xrn1 exoribonuclease, respectively. The 5′-NGD intermediate lacks a termination codon, and a ribosome translating a 5′-NGD intermediate may be stalled at the 3′ end. For a 5′-NGD intermediate to be rapidly degraded by the exosome, the stalled ribosome at the 3′ end of the mRNA fragment must be dissociated.

Dom34-Hbs1 has been proposed to be involved in the endonucleolytic cleavage of mRNA induced by blockage of translation elongation *in vivo*^6^,^7^,^9^,^10^,^11^. Recent biochemical analyses clearly demonstrated that yeast Dom34-Hbs1 promotes subunit dissociation and peptidyl-tRNA drop-off^12^,^13^. In yeast *in vitro* reconstituted translation system, Dom34-Hbs1 promotes the dissociation of the translation elongation complex into subunits, release of mRNA, and drop-off of peptidyl-tRNA^12^,^13^. Mammalian Pelota-human Hbs1 (hHbs1) also induces the dissociation of the translation elongation complex, but only in the presence of ABCE1, and Hbs1 has only a stimulatory effect^14^. A recent study showed that the yeast Dom34-Hbs1 complex dissociates a ribosome stalled at the 3′ end of a 5′-NGD intermediate and stimulates the exosomal degradation of the 5′-NGD intermediate *in vivo*^10^. The Nonstop Decay (NSD) quality control system eliminates nonstop mRNA, and Dom34-Hbs1 also facilitates the degradation of nonstop mRNAs from their 3′ ends by exosome^10^,^15^. These results indicate that the Dom34-Hbs1 complex plays a crucial role in both NGD and NSD by stimulating the degradation of this mRNA by dissociating the ribosome that is stalled at the 3′ end of nonstop mRNA. In *dom34*Δ mutant cells, a stalled ribosome at the 3′ end of nonstop mRNA may inhibit the degradation of nonstop mRNA by exosomes but induce sequential endonucleolytic cleavages. The mechanisms and physiological roles of sequential cleavages induced by stalled ribosome still remain elusive.

Nascent peptide-dependent translation arrest is crucial for quality control of eukaryotic gene expression. A genetic screen to identify factors required for induction of translation arrest by poly-arginine sequences revealed that *RACK1* (*ASC1* in yeast) is required for translation arrest^9^. Asc1/RACK1, which is highly conserved in eukaryotes, serves as a scaffold protein in many different signal transduction pathways. It also functions in developmental processes, such as sexual differentiation, cell-proliferation control, control of post-synaptic excitation in the brain and in hormone response pathways^16^,^17^,^18^,^19^. Asc1/RACK1 stably associates with the 40*S* subunit in a stoichiometric manner in yeast^20^,^21^,^22^. However, its role in translational control is still largely unknown. Two important questions to be resolved are how Asc1/RACK1 functions during translation arrest; how it can sense the properties of the nascent peptide in the ribosomal tunnel on the 60*S* subunit. Cryo-electron microscopy (Cryo-EM) structure of yeast ribosome revealed that the structure of the 60*S* subunit is affected by Asc1/RACK1 depletion^23^,^24^. Crystal structure studies showed that RACK1 binds to the head region of the 40*S* subunit in the vicinity of the mRNA exit channel^21^,^22^. These suggest that binding of Asc1/RACK1 to the 40*S* subunit stabilizes the conformation and/or the monitoring of the properties of nascent peptide in the ribosome tunnel by the 80*S* ribosome. It is also possible that recruitment of signaling factors through interactions with Asc1/RACK1 on the 40*S* subunit are required to modulate the activity of the ribosome or translation factors. A recent study showed that Asc1/RACK1 inhibits ribosomal +1 frameshifting at tandem CGA rare codons^25^. These findings suggest that Asc1/RACK1 may also modulate translation elongation at specific sites, implying that it plays an important and so far unappreciated role in the control of gene expression in eukaryotes.

Here, we describe the mechanism and biological relevance of a novel quality control system induced by stalled ribosomes at the 3′ end of mRNA. Asc1/RACK1 stimulated the degradation of truncated *GFP-Rz* mRNA that lacks both a termination codon and a poly(A) tail in *dom34*Δ*asc1*Δ mutant cells. The sequential endonucleolytic cleavages by stalled ribosomes at the 3′ end of mRNA were drastically decreased by the deletion of *ASC1/RACK1*. Small fragments of truncated *GFP-Rz* mRNA were purified with stalled ribosomes in an Asc1/Rack1 dependent manner. Overproduction of nonstop mRNA inhibited the growth of the *dom34*Δ*asc1*Δ mutant, possibly due to the elevated level of peptidyl-tRNA derived from truncated *GFP-Rz* mRNA. Moreover, the level of the peptidyl-tRNA was drastically increased by the deletion of Asc1/RACK1 in *dom34*Δ mutant background. Finally, we found that arrest products from truncated *GFP-Rz* mRNA in *asc1*Δ and *dom34*Δ mutant cells were degraded by Ltn1 that is an E3 ubiquitin ligase for co-translational degradation of arrest products. Based on our findings, we propose that Asc1/RACK1 stalls ribosome during aberrant translation elongation to induce endonucleolytic cleavages thereby reducing the level of aberrant mRNAs.

## Results

### Asc1/RACK1 facilitates the degradation of truncated *GFP-Rz* mRNA in the *dom34*Δ mutant background.

**Figure 1:**
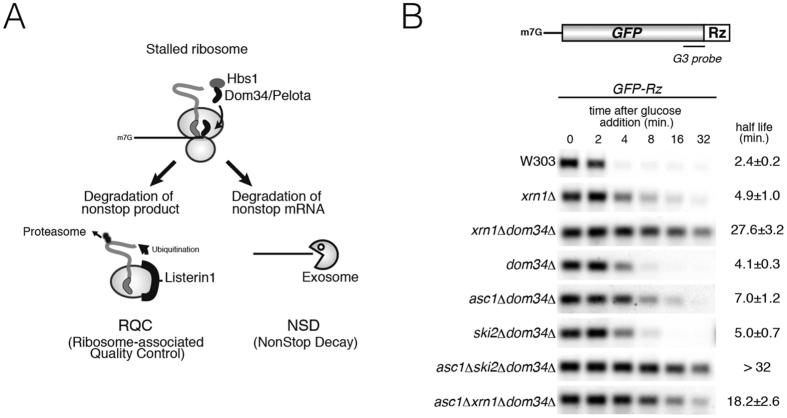
Asc1/RACK1 facilitates truncated *GFP-Rz* mRNA decay in the absence of 5′- to -3′ decay pathway. (**A**) Quality control of ribosome stalled at the 3′ end of truncated *GFP-Rz* mRNA. In the ribosome-associated quality control (RQC) system, a stalled ribosome dissociates into subunits by the functions of Dom34-Hbs1 in concert with Rli1, and the peptidyl-tRNA on the 60*S* subunit is ubiquitylated by Ltn1 Listerin in mammal to target it for proteasomal degradation. In NSD, Dom34-Hbs1 dissociates the stalled ribosome from nonstop mRNA, thereby facilitating degradation of nonstop mRNA by the exosome. (**B**) Asc1/RACK1 facilitates degradation of *GFP-Rz* mRNA in the absence of Dom34. (Top) Schematic drawing of truncated *GFP-Rz* mRNA derived from the *GFP-Rz-FLAG-HIS3* reporter gene. *GFP-Rz* mRNA, which lacks a poly(A) tail and a termination codon, is produced from *GFP-Rz-FLAG-HIS3* mRNA by the self-cleavage activity of hammerhead ribozyme (Rz). DIG-labeled *G3* probe corresponding to 654–714 nt of *GFP* is indicated. (Bottom) The stability of *GFP-Rz* mRNA in the indicated mutant cells. Cells were grown in SC-Ura media containing 2%w/v galactose, and transcription was inhibited by addition of 2%w/v glucose at mid-log phase. At the indicated times, cells were harvested, and the relative levels of truncated *GFP-Rz* mRNA were determined by Northern blot analysis using a DIG-labeled *G3* probe.

Previous work showed that Dom34-Hbs1 stimulates degradation of nonstop mRNA from the 3′ end^10^ (Fig. 1A). To determine whether Asc1/RACK1 is involved in NSD, we examined its role in the degradation of *GFP-Rz* mRNA in the absence of Dom34 (Fig. 1B). *GFP-Rz* mRNA is produced from *GFP-Rz-FLAG-HIS3* mRNA by the self-cleavage activity of hammerhead ribozyme(Rz), therefore this mRNA lacks a poly(A) tail and a termination codon. As previously reported, *GFP-Rz* mRNA was significantly more stable when Dom34 and Hbs1 were absent from the *xrn1*Δ mutant, in which only the 3′-to-5′ degradation pathway is active^10^ (Fig. 1B, *t*_1/2_ = 4.9 min. for *xrn1*Δ vs. *t*_1/2_ = 27.6 min. for *xrn1*Δ*dom34*Δ). We found that Asc1/RACK1 destabilized *GFP-Rz* mRNA in the absence of Dom34 (Fig. 1B, *t*_1/2_ = 4.1 min. for *dom34*Δ vs. *t*_1/2_ = 7.0 min. for *dom34*Δ*asc1*Δ). Asc1/RACK1 also destabilized *GFP-Rz* mRNA in *ski2*Δ*dom34*Δ mutant background (Fig. 1B, *t*_1/2_ = 5.0 min. for *ski2*Δ*dom34*Δ vs. *t*_1/2_ > 32 min. for *asc1*Δ*ski2*Δ*dom34*Δ). These indicate that Asc1/RACK1 facilitates truncated *GFP-Rz* mRNA decay in the absence of exosome-dependent decay and Dom34.

### Asc1/RACK1 is required for endonucleolytic cleavage of truncated *GFP-Rz* mRNA in the *dom34*Δ mutants.

**Figure 2:**
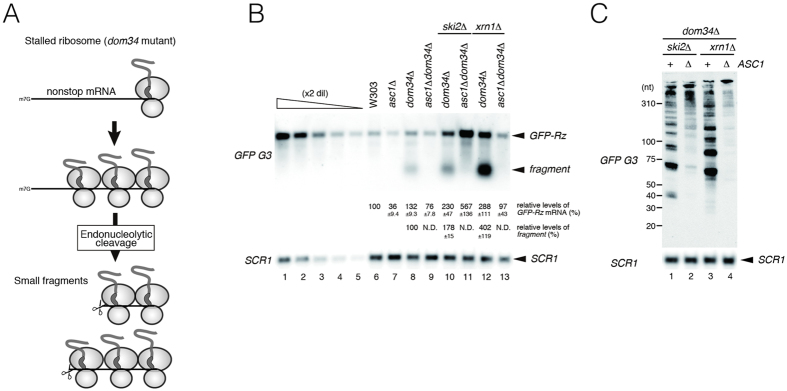
Asc1/RACK1 is required for sequential endonucleolytic cleavages induced by stalled ribosomes. (**A**) Multiple endonucleolytic cleavages of truncated *GFP-Rz* mRNA in the *dom34*Δ mutant background. (**B**) Asc1/RACK1 is required for multiple endonucleolytic cleavages of *GFP-Rz* mRNA induced by stalled ribosomes. RNA samples were prepared from the indicated cells harboring p416*GAL1p-GFP-Rz-FLAG-HIS3* and analyzed by 1.2% agarose and Northern blotting using DIG-labeled *G3* and *SCR1* probes. The levels of *GFP-Rz* mRNA and fragments were determined using the samples from *ski2*Δ*dom34*Δ*asc1*Δ cells harboring p416*GPDp-GFP-Rz-FLAG-HIS3* as a standard curve (lanes 1–5). The relative levels of each mRNA or fragments were normalized with respect to the *GFP-Rz* mRNA level in wild-type cells, which was assigned a value of 100, and reported as the mean values from three independent experiments. (**C**) Asc1/RACK1 is required for multiple endonucleolytic cleavages of *GFP-Rz* mRNA induced by stalled ribosomes. RNA samples were prepared from the indicated cells harboring p416*GAL1p-GFP-Rz-FLAG-HIS3* and analyzed by 6% TBE-Urea PAGE and Northern blotting using DIG-labeled *G3* and *SCR1* probes.

We previously reported that Asc1/RACK1 is involved in NGD, endonucleolytic cleavage induced by translation arrest^9^. Therefore, we hypothesized that sequential endonucleolytic cleavages destabilize *GFP-Rz* mRNA in *ski2*Δ*dom34*Δ mutant cells, and Asc1/RACK1 is required for the sequential endonucleolytic cleavages induced by the stalled ribosome at the 3′ end of *GFP-Rz* mRNA in *ski2*Δ*dom34*Δ mutant cells. To test the possibility that Asc1/RACK1 is required for the sequential endonucleolytic cleavages induced by the stalled ribosome, we determined the levels of 40–200 nt fragments derived from *GFP-Rz* reporter mRNAs by Northern blot analysis, as previously described^10^ (Fig. 2A). Upon deletion of *ASC1*/*RACK1*, the levels of fragments derived from *GFP-Rz* mRNA were drastically reduced in *dom34*Δmutant cells (Fig. 2B, lanes 8 and 9), *ski2*Δ*dom34*Δ mutant cells (Fig. 2B, lanes 10 and 11) or *xrn1*Δ*dom34*Δ mutant cells (Fig. 2B, lanes 12 and 13). Deletion of *ASC1*/*RACK1* diminished the production of fragments in *ski2*Δ*dom34*Δ and *xrn1*Δ*dom34*Δ mutant cells (Fig. 2C). These findings indicate that Asc1/RACK1 is generally required for the sequential endonucleolytic cleavages induced by stalled ribosomes at the 3′ ends of mRNA.

### Small fragments of truncated *GFP-Rz* mRNA were purified with stalled ribosomes in an Asc1/Rack1 dependent manner.

**Figure 3:**
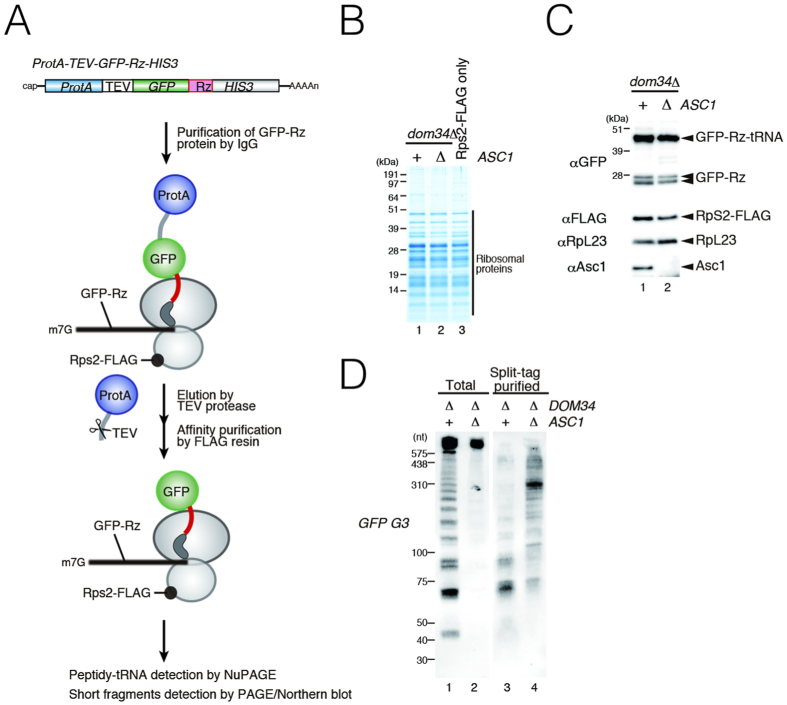
Purification of stalled ribosome at the 3′ end of *GFP-Rz* truncated mRNA. (**A**) Schematic illustration of the split-tag affinity purification of the stalled ribosome. (**B**) Cells harboring p*GPDp-ProteinA-TEV-GFP-Rz-HIS3* were harvested and cell lysates prepared for split-tag purification. Protein samples of the purified fractions were analyzed by CBB stain (lanes 1–2). Ribosomes were affinity-purified from Rps2-FLAG cells as a control (lane 3). (**C**) Protein samples of the purified fractions were analyzed by NuPAGE followed by Western blot analysis with anti-GFP antibody (Top panel) or anti-FLAG, anti-RpL23 and anti-Asc1 antibodies (Bottom panles). (**D**) RNA samples of the purified fractions were analyzed by Northern blotting using DIG-labeled *G3* probes after PAGE.

To confirm that stalled ribosome at the 3′ end of mRNA in *dom34*Δ mutant cells contains small RNA fragments, we purified stalled ribosomes from cells expressing both Protein A-TEV-GFP-Rz and Rps2-FLAG proteins as depicted in Fig. 3A. The Protein A-TEV-GFP-Rz truncated products derived from *GFP-Rz* truncated mRNA were purified with IgG affinity beads and eluted by the addition of TEV protease. Then stalled ribosomes were affinity-purified with anti-FLAG resin. The stalled ribosomes containing peptidyl-tRNA were purified from both *dom34*Δ mutant and *asc1*Δ*dom34*Δ mutant cells with this (Fig. 3B,C). Northern blot analysis of the purified samples with *GFP* G3 probe clearly revealed that the purified stalled ribosomes from *dom34*Δ cells contain fragments derived from *GFP-Rz* mRNA (Fig. 3D, lane 3). In contrast, stalled ribosomes from *asc1*Δ*dom34*Δ cells contain less amounts of small fragments derived from *GFP-Rz* mRNA, and the levels of fragments were the same as that from *dom34*Δ mutant cells (Fig. 3D). These results clearly indicate that stalled ribosomes at the 3′ end of *GFP-Rz* mRNA in *dom34*Δ mutant cells contain fragments produced by sequential endonucleolytic cleavages in an Asc1/RACK1-dependent manner.

### An endonucleolytic cleavage induced by stalled ribosome requires ribosome-associated Asc1/RACK1

**Figure 4:**
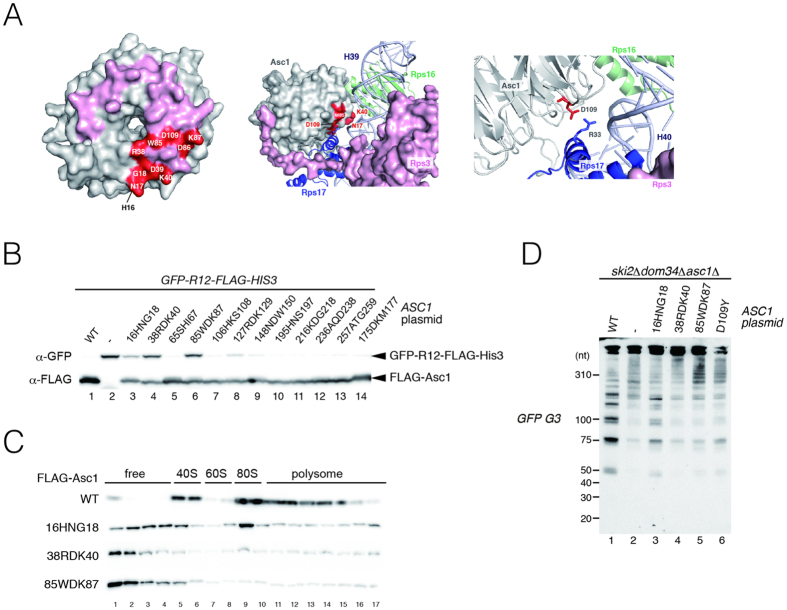
Ribosome binding activity of Asc1/RACK1 is required for sequential endonucleolytic cleavages induced by stalled ribosomes. (**A**) The structure diagram was prepared in PyMOL using PDB file 4V88. (Left) Residues located in the putative interaction sites of Asc1/RACK1 with the 40*S* subunit are shown in pink. Residues replaced with alanine residues in mutants defective in translation arrest by polybasic sequence and the interaction with 40S subunit were shown in Red. (Middle) The residues involved in translation arrest by polybasic sequence and the interaction with 40S subunit locate close to H39-H40 and Rps17. (Right) Relative position of D109 of Asc1 to R33 of Rps17. (**B**) The ribosome binding of Asc1/RACK1 mutant proteins defective in translation repression by polybasic amino-acid sequence. The *asc1*Δ cells harboring the *GFP-R12-FLAG-HIS3* reporter were transformed with the various FLAG-Asc1/RACK1 plasmids. The levels of the *GFP-R12-FLAG-HIS3* reporter proteins were determined by Western blot analysis with anti-GFP and anti-FLAG antibodies. (**C**) Ribosome binding of Asc1/RACK1 mutant proteins. Cell extracts were prepared from the *asc1*Δ cells expressing various FLAG-Asc1/RACK1 mutant proteins. Extracts were subjected to polysome analysis, followed by Western blot analysis with anti-FLAG antibody. (**D**) Ribosome-associated Asc1/RACK1 is required for multiple endonucleolytic cleavages of *GFP-Rz* mRNA in a *dom34*Δ mutant background. *ski2*Δ*dom34*Δ*asc1*Δ mutant cells harboring p416*GAL1p-GFP-Rz-FLAG-HIS3* were transformed with the indicated plasmids expressing Asc1/RACK1 mutants. RNA samples were analyzed by 6% TBE-Urea PAGE and Northern blotting using DIG-labeled *G3* probes.

We previously reported that Asc1/RACK1 participates in nascent peptide-dependent translation arrest, and that the binding of Asc1/RACK1 to the 40S subunit is crucial for this translation arrest. The *asc1*D109Y and *asc1*R38DK40E mutants were found to produce severe defects both in translation repression by polybasic sequence and in the association between RACK1 and the ribosome^9^. We introduced mutations in residues located at the sites of interaction of Asc1/RACK1 with the 40*S* subunit^26^ (Fig. 4A, pink residues). We determined the levels of the full-length products derived from *GFP-R12-FLAG-HIS3* (R12) reporter, and three Asc1/RACK1 mutant proteins (16HNG18AAA, 38RDK40AAA and 85WDK87AAA) were defective in translation arrest induced by a polybasic amino-acid sequence (Fig. 4B). Western blot analysis followed by centrifugation in sucrose density gradients revealed that these three Asc1/RACK1 mutant proteins (16HNG18AAA, 38RDK40AAA and 85WDK87AAA) were defective in interaction with ribosome (Fig. 4C). These results are consistent with previous results showing that the binding of Asc1/RACK1 to the 40S subunit is crucial for translation arrest induced by polybasic sequence^9^. We next examined whether the 40S subunit binding of Asc1/RACK1 is crucial for endonucleolytic cleavage induced by stalled ribosome at the 3′ end of nonstop mRNA. Production of fragments was diminished in *ski2*Δ*dom34*Δ and *xrn1*Δ*dom34*Δ mutant cells expressing these Asc1/RACK1 mutants (Fig. 4D). These indicate that the Asc1/RACK1-bound ribosomes are competent to induce the sequential endonucleolytic cleavages induced by stalled ribosomes at the 3′ end of mRNA.

### Overproduction of truncated *GFP-Rz* mRNAs inhibits cell growth of the *ski2*Δ*dom34*Δ*asc1*Δ mutant.

**Figure 5:**
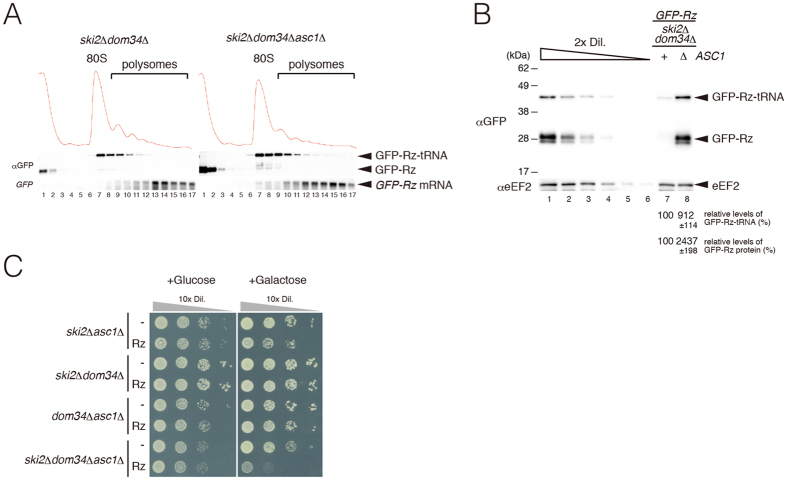
Asc1/RACK1 inhibits protein production from nonstop mRNA in the absence of Dom34. (**A**) Cell extracts were prepared from the indicated cells harboring p416*GPDp-GFP-Rz-FLAG-HIS3* plasmid, and polysome analysis was performed. (Top) Absorbance at 254 nm. (Middle) Neutral-PAGE followed by Western blotting with anti-GFP antibodies. (Bottom) Northern blotting using DIG-labeled *G3* probes. (**B**) Levels of peptidyl-tRNA derived from *GFP-Rz* mRNA in *ski2*Δ*dom34*Δ or *ski2*Δ*dom34*Δ*asc1*Δ mutant cells. Cell extracts were analyzed by Neutral-PAGE, followed by Western blotting using anti-GFP and anti-eEF2 antibodies. The levels of GFP-Rz and peptidyl-tRNA were determined using the samples from *ski2*Δ*dom34*Δ*asc1*Δ cells harboring p416*GPDp-GFP-Rz-FLAG-HIS3* as a standard curve (lanes 1–6). The relative levels of each GFP-Rz and peptidyl-tRNAs were normalized with respect to the levels of GFP-Rz and peptidyl-tRNAs in wild-type cells, which was assigned as a value of 100, and reported as the mean values from three independent experiments. (**C**) Overproduction of aberrant mRNAs lacking a termination codon inhibits growth of the *ski2*Δ*dom34*Δ*asc1*Δ mutant. Cells harboring p*GAL1p-GFP-Rz-FLAG-HIS3* were grown in SC-Ura media containing 2% raffinose to log-phase, and the cells in a series of dilutions were grown on SC-Ura plates containing 2% glucose or 2% galactose.

Dom34-Hbs1 promotes dissociation of the translation elongation complex into subunits, and we detected peptidyl-tRNA derived from *GFP-Rz* mRNA in cell extracts of the *ski2*Δ*dom34*Δ mutant^10^. Although *GFP-Rz* mRNAs were mainly distributed in heavy polysome fractions, the peptidyl-tRNA is only present in monosome or light polysome fractions^10^. Because the sequential endonucleolytic cleavages were almost abolished in the absence of *ASC1*/*RACK1*, we had expected to detect the peptidyl-tRNA derived from *GFP-Rz* mRNA in heavy polysome fractions derived from cell extracts of the *ski2*Δ*dom34*Δ*asc1*Δ mutant. However, in the *ski2*Δ*dom34*Δ*asc1*Δ mutant, the peptidyl-tRNA derived from *GFP-Rz* mRNA was distributed in the monosome and light polysome fractions, and the distribution of peptidyl-tRNA was almost the same as that in *ski2*Δ*dom34*Δ mutant cells (Fig. 5A). The levels of peptidyl-tRNA in polysome fractions and the polypeptide in ribosome-free fractions were significantly increased by *ASC1*/*RACK1* deletion in the *ski2*Δ*dom34*Δ mutant background (Fig. 5A). Therefore, we quantified the levels of peptidyl-tRNA and free polypeptide derived from *GFP-Rz* mRNA in cell lysates (Fig. 5B). Neutral-PAGE (NuPAGE) followed by Western blot analysis revealed that the levels of peptidyl-tRNA and free polypeptide derived from *GFP-Rz* mRNA were drastically increased by *ASC1*/*RACK1* deletion in the *ski2*Δ*dom34*Δ mutant background. The level of peptidyl-tRNA in *ski2*Δ*dom34*Δ*asc1*Δ mutant was 9-fold higher than that in *ski2*Δ*dom34*Δ mutant cells, and the level of free polypeptide GFP-Rz in the *ski2*Δ*dom34*Δ*asc1*Δ mutant was 24-fold higher than that in *ski2*Δ*dom34*Δ mutant cells (Fig. 5B). These findings indicate that nonstop protein may be synthesized from *GFP-Rz* mRNA even in the absence of the Dom34-Hbs1 complex. Therefore, we propose that ribosomes stalled at the 3′ end of mRNA in the absence of Dom34-Hbs1 may be dissociated into subunits in the *asc1*Δ mutant background. To determine the biological significance of this novel quality control system, we examined the effects of overproduction of aberrant mRNAs without termination codons in both *ski2*Δ*dom34*Δ*asc1*Δ mutant and *ski2*Δ*dom34*Δ mutant. To overproduce *GFP-Rz* mRNA, cells harboring p*GAL1p-GFP-Rz-FLAG-HIS3* plasmid were grown on SC-Ura plates containing galactose. Overproduction of *GFP-Rz* nonstop mRNA inhibited growth of the *ski2*Δ*dom34*Δ*asc1*Δ mutant, but not the *ski2*Δ*dom34*Δ mutant (Fig. 5C). We propose that when the quality control system by Dom34-Hbs1 is impaired, Asc1/RACK1 plays a crucial role in the repression of aberrant products derived from mRNAs lacking termination codons by inducing sequential endonucleolytic cleavages.

### Arrest products from truncated *GFP-Rz* mRNA were subjected to Ltn1-dependent degradation in *asc1* and *dom34* mutant cells.

**Figure 6:**
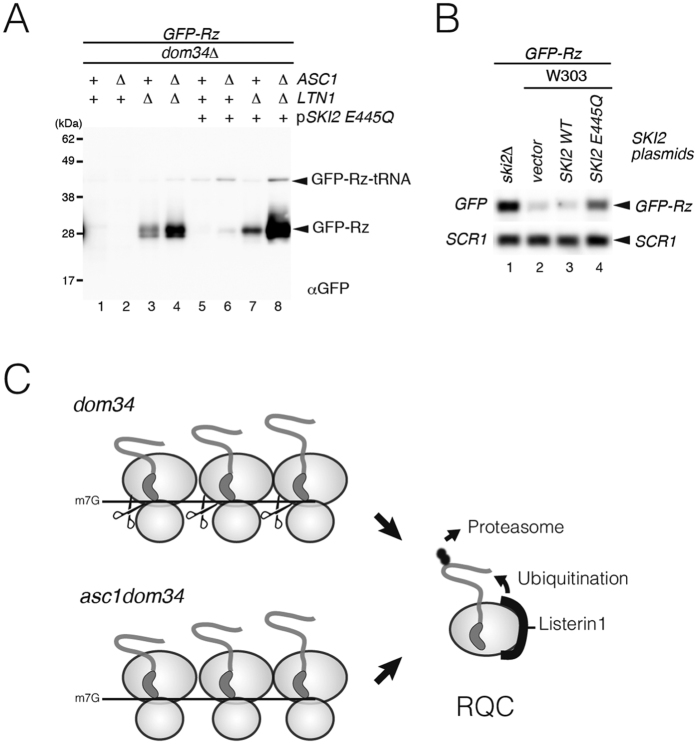
Nonstop products derived from truncated *GFP-Rz* mRNA were degraded by RQC in *dom34* and *dom34asc1* mutant cells. (**A**) Levels of peptidyl-tRNA and GFP-Rz protein derived from *GFP-Rz* mRNA were significantly increased by the deletion of Ltn1 in *dom34*Δ or *dom34*Δ*asc1*Δ mutant cells. Cell extracts prepared from the indicated cells containing *GFP-Rz-FLAG-HIS3* reporter were analyzed by Neutral-PAGE, followed by Western blotting using anti-GFP antibody. When indicated, cells were transformed with Ski2 E445Q plasmid (Lanes 5–8). (**B**) The level of truncated *GFP-Rz* mRNA was increased in wild type cells in the presence of dominant-negative Ski2-E445Q protein. (**C**) A model for quality control of ribosomes stalled at 3′ ends of nonstop mRNAs in *dom34*Δ and *dom34*Δ*asc1*Δ mutant. (Top) In the absence of Dom34, a stalled ribosome at the 3′ end of a nonstop mRNA induces sequential cleavage, resulting in production of a ladder of small fragments protected by ribosomes. (Bottom) In the absence of Dom34 and Asc1/RACK1, a ribosome reaching the 3′ end of a nonstop mRNA dissociates from mRNA as an 80*S* particle containing peptidyl-tRNA. In both mutant cells, Ltn1 degrades nonstop products derived from *GFP-Rz* mRNA.

Co-translational degradation of polypeptide nascent chains plays a critical role in quality control of protein synthesis and the rescue of stalled ribosomes. Nonstop proteins are recognized by the ribosome-associated quality control (RQC) machinery, and targeted for proteasomal degradation^27^,^28^,^29^,^30^,^31^,^32^. Dom34-Hbs1 and its mammalian homolog Pelota-hHbs1 is involved in RQC by facilitating the dissociation of stalled ribosomes at the 3′ end of mRNA^32^. We previously reported that the level of the protein products derived from the reporter gene containing poly-lysine sequences within the ORF was drastically increased in *asc1*Δ mutant, whereas the levels of arrest products were significantly decreased in *asc1*Δ mutant even in the presence of the proteasome inhibitor^9^. Therefore, we determined the levels of GFP-Rz and the peptidyl-tRNA in *dom34*Δ and *dom34*Δ*asc1*Δ mutant cells. Ltn1 significantly decreased the levels of Nonstop products in *dom34*Δmutant cells (Fig. 6A, lanes 1 and 3) and *dom34*Δ*asc1*Δ mutant cells (Fig. 6A, lanes 2 and 4). The ATPase activity of the Ski2-3-8 complex was abolished by E445Q mutation in the Ski2 DExH core^33^. The level of *GFP-Rz* mRNA was increased in the presence of Ski2-E445Q mutant protein in wild type cells (Fig. 6B). Because of the problems during the construction of *ski2*Δ*asc1*Δ*ltn1*Δ*dom34*Δ tetra mutants, we introduced the dominant negative Ski2-E445Q mutant to inhibit the exosome activity^33^. In the presence of Ski2-E445Q mutant protein, Ltn1 also decreased the levels of GFP-Rz and the peptidyl-tRNA in *dom34*Δ and *dom34*Δ*asc1*Δ mutant cells (Fig. 6A, lanes 5–8). These indicate that Ltn1 degrades the arrest products from truncated *GFP-Rz* mRNA even in the absence of Asc1 and Dom34.

## Discussion

The Dom34-Hbs1 complex dissociates ribosomes stalled at the 3′ ends of 5′-NGD intermediates and nonstop mRNAs and stimulates their degradation by the exosome^10^. In *dom34*Δ mutant cells, such stalled ribosomes induce sequential endonucleolytic cleavages^10^. These cleavages have been proposed to play novel roles in NGD and NSD by reducing the levels of 5′-NGD intermediates or nonstop mRNA in the absence of Dom34-Hbs1-dependent mRNA decay mediated by the exosome^10^. The results in this study clearly showed that Asc1/RACK1 is required for sequential endonucleolytic cleavages induced by stalled ribosome at the 3′ end of truncated *GFP-Rz* mRNA. Asc1/RACK1 also was reported to facilitate the endonucleolytic cleavages induced by translation arrests by polybasic sequences^9^. Therefore we propose that Asc1/RACK1 is a quality control factor to stimulate the endonucleolytic cleavages by stalled ribosome generally.

The level of *GFP-Rz* mRNA in *ski2*Δ*dom34*Δ*asc1*Δ mutant was 2.5-fold higher than that in *ski2*Δ*dom34*Δmutant cells (Fig. 2B). The levels of peptidyl-tRNA and GFP-Rz derived from truncated *GFP-Rz* mRNA were increased more than 9-fold and 24-fold respectively, by *ASC1*/*RACK1* deletion in the *ski2*Δ*dom34*Δ mutant background (Fig. 5B). Therefore, translation efficiency based on the ratio between protein products and mRNA levels was significantly increased by *ASC1*/*RACK1* deletion in the *ski2*Δ*dom34*Δ mutant background. Moreover, Asc1/RACK1 moderately but significantly stabilized *GFP-Rz* mRNA in *xrn1*Δ*dom34*Δ mutant background (Fig. 1B, *t*_1/2_ = 27.6 min. for *xrn1*Δ*dom34*Δ vs. *t*_1/2_ = 18.2 min. for *asc1*Δ*xrn1*Δ*dom34*Δ), suggesting that Asc1/RACK1 facilitates the exosome-dependent decay from 3′ end of *GFP-Rz* mRNA. We suspect that ribosomes reaching the 3′ end of a nonstop mRNA rapidly dissociate as 80S particles containing peptidyl-tRNA in the absence of Dom34 and Asc1/RACK1, resulting in elevated levels of peptidyl-tRNA derived from nonstop mRNA. We previously reported that Asc1/RACK1 is required for translation arrest induced by polybasic amino-acid sequence or tandem rare codons^9^,^34^. Taken together, these observations suggest that Asc1/RACK1 plays general roles in translation regulation and mRNA quality control systems, and that Asc1/RACK1-bound ribosomes have the potential to stall in response to specific nascent peptide sequences, low levels of aminoacyl-tRNA, or a lack of a codon at the A-site.

Recent study showed that Asc1 promotes translation of mRNAs with short open reading frames^35^. The D109Y mutant showed translational defects that, although correlated with those observed in the *asc1*Δ mutants were much smaller in magnitude. The R38DK40E mutant showed almost negligible effects on translation^35^. In contrast, the defects of Asc1 mutants (38RDK40AAA, 85WDK87AAA, D109Y) in translation arrest by polybasic sequence and the endonucleolytic cleavage induced by stalled ribosome were almost the same as that of the *asc1*Δ mutant, although 16HNG18AAA mutant showed moderate effects (Fig. 4). We propose that robust and proper interaction between Asc1/RACK1 with 40S subunit may be crucial for co-translational quality controls to induce translation elongation or mRNA cleavage, but dispensable to promote translation of mRNAs with short open reading frames. Drosophila RACK1 is required in the 40S ribosomal subunit for IRES-dependent translation of Cricket Paralysis Virus and the picorna-like Drosophila C Virus^36^. D108Y and R38D/K40E mutants were defective in these virus RNAs^36^, suggesting the regulatory roles of the interaction of Asc1/RACK1 with 40S subunit. The identification of the interacting partner for Asc1/RACK1 is crucial to elucidate mechanisms of RACK1-dependent regulation of translation or mRNA decay.

The results in this study clearly showed that Ltn1 destabilizes aberrant nonstop polypeptides even in the absence of the ribosome dissociation factor Dom34:Hbs1 as previously reported^34^. We also found that Ltn1 destabilizes nonstop products derived from *GFP-Rz* mRNA in the absence of ASC1/RACK1. These indicate that ASC1/RACK1 is dispensable for RQC for nonstop products. Previous results showed that Ltn1 destabilizes arrest products derived from *GFP-R12-FLAG-HIS3* reporter in a RACK1-dependent manner^34^, indicating that the roles of Asc1/RACK1 in RQC differs depending on where ribosomes stalled. Mechanisms of the dissociation of stalled ribosomes are still largely unknown, and future study will identify putative factors that recognize stalled ribosome and induce the subunit dissociation independent of Dom34-Hbs1.

## Methods

### Strains and other methods

The strains and plasmids used in this study are listed in Supplementary Table 1. Northern blot analysis, protein preparation, Western blot analysis were performed as previously described^10^,^27^.

### Yeast extract and sucrose gradient separation

Yeast cells were grown exponentially at 30 °C and harvested by centrifugation. Cell extracts were prepared as described previously^20^. The equivalent of 50 A260 units were layered onto linear 10–50% sucrose density gradients. Sucrose gradients (10–50% sucrose in 10 mM Tris-acetate pH 7.4, 70 mM ammonium acetate, and 4 mM magnesium acetate) were prepared in polyallomer tubes (Beckman Coulter) using a Gradient Master. Crude extracts were layered on top of the sucrose gradients and centrifuged at 150,000 × *g* in a P28S rotor (Hitachi Koki, Japan) for 3 hours at 4 °C. Gradients were then fractionated (TOWA lab, Tsukuba). Polysome profiles were generated by continuous absorbance measurement at 254 nm using a single path UV-1 optical unit (ATTO BioMini UV-monitor) connected to a chart recorder (ATTO digital mini-recorder). Equal volume fractions were collected and processed for Northern or Western blotting as described above^37^.

### Determination of mRNA stability

Yeast cells were grown in minimal medium (SC medium) containing 2% galactose. Cells were grown to OD600 = 0.6, harvested and resuspended in medium containing 2% glucose to inhibit transcription from the *GAL1* promoter. At the indicatedtimes, the cells were harvested to prepare RNA samples by acidic phenol RNA extraction method using water-saturated phenol and phenol-chloroform-isoamylalcohol (25:24:1) mixture. mRNA levels of reporter genes were determined by Northern blotting using digoxigenin (DIG)-labeled *GFP* probes prepared by PCR-based nucleic acid labeling using PCR DIG Probe Synthesis kit (Roche, NJ, USA) according to the procedure specified by the manufacturer and primers 5′-GCTCTAGAATGAGTAAAGGAGAAGAACTTTTCAC-3′ and 5′-GGACTAGTTTTGTATAGTTCATCCATGCCA-3′. *GFP G3* probe is a 5′ end DIG-labeled oligonucleotide 5′-TTTGTATAGTTCATCCATGCCATGTGTAATCCCAGCAGCAGTTACAAACTCAAGAAGGACC-3′. The intensity of bands on the blots was quantified on a LAS4000 mini (GE Healthcare). Relative RNA levels were determined using Multi Gauge v3.0 (Fujifilm, Japan) by comparison to a standard curve using a series of dilutions of samples from time 0 (just before the addition of glucose).

### Split-tag affinity purification of stalled ribosomes

Stalled ribosomes were purified from cells expressing both ProtA-TEV-GFP-Rz-His3 containing and Rps2-FLAG proteins by split-tag affinity purification. Yeast cells were transformed with plasmids harbouring *ProteinA-TEV-GFP-Rz-HIS3* gene were cultured in 1 L of synthetic complete medium. The ProteinA-TEV-GFP-Rz was affinity-purified from whole cell lysates using IgG Sepharose^TM^ (GE Healthcare), followed by the treatment with TEV protease. Ribosome complex containing ProteinA-TEV-GFP-Rz was purified with Anti-DYKDDDDK tag antibody beads (Wako, Japan) and eluted by 250ng/μL of FLAG peptides.

### Detection of peptidyl-tRNA

Cell extracts or polysome fractions were analyzed by NuPAGE (Invitrogen, USA) followed by Western blotting with anti-GFP antibodies^10^. To destroy the RNA moiety of peptidyl-tRNA, RNase A was added to the samples at a final concentration of 10 mg/*l* and the samples were incubated at 37 °C for 10 min. (data not shown).

### Spot assay

Cells harboring p416*GAL1p-GFP-Rz-FLAG-HIS3* plasmids were grown with SC-Ura media containing 2% raffinose to OD600 = 0.3. The cells were spotted on SC-Ura plate containing 2% glucose or 2% galactose after a series of 10-fold dilution, and incubated at 30 °C for 2 days (glucose) or 3 days (galactose).

### Overexpression of dominant-negative Ski2 and inhibition of exosome

The ATPase activity of the Ski2-3-8 complex was abolished by E445Q mutation in the Ski2 DExH core^33^. To inhibit exosome activity, p415*GPDp-SKI2 E445Q* plasmids were introduced to cells. Inhibition of exosome was confirmed by northern blot to detect *GFP-Rz* mRNA.

## Additional Information

**How to cite this article**: Ikeuchi, K. and Inada, T. Ribosome-associated Asc1/RACK1 is required for endonucleolytic cleavage induced by stalled ribosome at the 3′ end of nonstop mRNA. *Sci. Rep.* **6**, 28234; doi: 10.1038/srep28234 (2016).

## Supplementary Material

Supplementary Information

## References

[b1] Schoenberg D. R. & Maquat L. E. Regulation of cytoplasmic mRNA decay. Nat Rev Genet 13, 246–259, doi: 10.1038/nrg3160 (2012). 22392217 PMC3351101

[b2] Pechmann S., Willmund F. & Frydman J. The ribosome as a hub for protein quality control. Mol Cell 49, 411–421, doi: 10.1016/j.molcel.2013.01.020 (2013). 23395271 PMC3593112

[b3] Shao S. & Hegde R. S. Target Selection during Protein Quality Control. Trends Biochem Sci 41, 124–137, doi: 10.1016/j.tibs.2015.10.007 (2016). 26628391

[b4] Brandman O. & Hegde R. S. Ribosome-associated protein quality control. Nat Struct Mol Biol 23, 7–15, doi: 10.1038/nsmb.3147 (2016). 26733220 PMC4853245

[b5] Lykke-Andersen J. & Bennett E. J. Protecting the proteome: Eukaryotic cotranslational quality control pathways. J Cell Biol 204, 467–476, doi: 10.1083/jcb.201311103 (2014). 24535822 PMC3926952

[b6] Doma M. K. & Parker R. Endonucleolytic cleavage of eukaryotic mRNAs with stalls in translation elongation. Nature 440, 561–564, doi: 10.1038/nature04530 (2006). 16554824 PMC1839849

[b7] Passos D. O. *et al.* Analysis of Dom34 and its function in no-go decay. Mol Biol Cell 20, 3025–3032, doi: 10.1091/mbc.E09-01-0028 (2009). 19420139 PMC2704154

[b8] Harigaya Y. & Parker R. No-go decay: a quality control mechanism for RNA in translation. Wiley Interdiscip Rev RNA 1, 132–141, doi: 10.1002/wrna.17 (2010). 21956910

[b9] Kuroha K. *et al.* Receptor for activated C kinase 1 stimulates nascent polypeptide-dependent translation arrest. EMBO Rep 11, 956–961, doi: 10.1038/embor.2010.169 (2010). 21072063 PMC2999862

[b10] Tsuboi T. *et al.* Dom34:hbs1 plays a general role in quality-control systems by dissociation of a stalled ribosome at the 3′ end of aberrant mRNA. Mol Cell 46, 518–529, doi: 10.1016/j.molcel.2012.03.013 (2012). 22503425

[b11] van den Elzen A. M. *et al.* Dissection of Dom34-Hbs1 reveals independent functions in two RNA quality control pathways. Nat Struct Mol Biol 17, 1446–1452, doi: 10.1038/nsmb.1963 (2010). 21102444

[b12] Shoemaker C. J., Eyler D. E. & Green R. Dom34:Hbs1 promotes subunit dissociation and peptidyl-tRNA drop-off to initiate no-go decay. Science 330, 369–372, doi: 10.1126/science.1192430 (2010). 20947765 PMC4022135

[b13] Shoemaker C. J. & Green R. Kinetic analysis reveals the ordered coupling of translation termination and ribosome recycling in yeast. Proc Natl Acad Sci USA 108, E1392–1398, doi: 10.1073/pnas.1113956108 (2011). 22143755 PMC3251084

[b14] Pisareva V. P., Skabkin M. A., Hellen C. U., Pestova T. V. & Pisarev A. V. Dissociation by Pelota, Hbs1 and ABCE1 of mammalian vacant 80S ribosomes and stalled elongation complexes. EMBO J 30, 1804–1817, doi: 10.1038/emboj.2011.93 (2011). 21448132 PMC3101999

[b15] Saito S., Hosoda N. & Hoshino S. I. Hbs1-Dom34 functions in non-stop mRNA decay (NSD) in mammalian cells. J Biol Chem, doi: 10.1074/jbc.M112.448977 (2013). PMC3682582 23667253

[b16] Guo J. *et al.* Dissection of the relationship between RACK1 and heterotrimeric G-proteins in Arabidopsis. Plant Cell Physiol 50, 1681–1694, doi: 10.1093/pcp/pcp113 (2009). 19651700

[b17] Volta V. *et al.* RACK1 depletion in a mouse model causes lethality, pigmentation deficits and reduction in protein synthesis efficiency. Cell Mol Life Sci 70, 1439–1450, doi: 10.1007/s00018-012-1215-y (2013). 23212600 PMC11113757

[b18] Yaka R. *et al.* NMDA receptor function is regulated by the inhibitory scaffolding protein, RACK1. Proc Natl Acad Sci USA 99, 5710–5715, doi: 10.1073/pnas.062046299 (2002). 11943848 PMC122836

[b19] Kadrmas J. L., Smith M. A., Pronovost S. M. & Beckerle M. C. Characterization of RACK1 function in Drosophila development. Dev Dyn 236, 2207–2215, doi: 10.1002/dvdy.21217 (2007). 17584887

[b20] Inada T. *et al.* One-step affinity purification of the yeast ribosome and its associated proteins and mRNAs. RNA 8, 948–958 (2002). 12166649 10.1017/s1355838202026018 PMC1370311

[b21] Coyle S. M., Gilbert W. V. & Doudna J. A. Direct link between RACK1 function and localization at the ribosome *in vivo*. Mol Cell Biol 29, 1626–1634, doi: 10.1128/MCB.01718-08 (2009). 19114558 PMC2648249

[b22] Rabl J., Leibundgut M., Ataide S. F., Haag A. & Ban N. Crystal structure of the eukaryotic 40S ribosomal subunit in complex with initiation factor 1. Science 331, 730–736, doi: 10.1126/science.1198308 (2011). 21205638

[b23] Nilsson J., Sengupta J., Frank J. & Nissen P. Regulation of eukaryotic translation by the RACK1 protein: a platform for signalling molecules on the ribosome. EMBO Rep 5, 1137–1141, doi: 10.1038/sj.embor.7400291 (2004). 15577927 PMC1299186

[b24] Sengupta J. *et al.* Identification of the versatile scaffold protein RACK1 on the eukaryotic ribosome by cryo-EM. Nat Struct Mol Biol 11, 957–962, doi: 10.1038/nsmb822 (2004). 15334071

[b25] Wolf A. S. & Grayhack E. J. Asc1, homolog of human RACK1, prevents frameshifting in yeast by ribosomes stalled at CGA codon repeats. RNA 21, 935–945, doi: 10.1261/rna.049080.114 (2015). 25792604 PMC4408800

[b26] Taylor D. J. *et al.* Comprehensive molecular structure of the eukaryotic ribosome. Structure 17, 1591–1604, doi: 10.1016/j.str.2009.09.015 (2009). 20004163 PMC2814252

[b27] Ito-Harashima S., Kuroha K., Tatematsu T. & Inada T. Translation of the poly(A) tail plays crucial roles in nonstop mRNA surveillance via translation repression and protein destabilization by proteasome in yeast. Genes Dev 21, 519–524, doi: 10.1101/gad.1490207 (2007). 17344413 PMC1820893

[b28] Dimitrova L. N., Kuroha K., Tatematsu T. & Inada T. Nascent peptide-dependent translation arrest leads to Not4p-mediated protein degradation by the proteasome. J Biol Chem 284, 10343–10352, doi: 10.1074/jbc.M808840200 (2009). 19204001 PMC2667721

[b29] Brandman O. *et al.* A ribosome-bound quality control complex triggers degradation of nascent peptides and signals translation stress. Cell 151, 1042–1054, doi: 10.1016/j.cell.2012.10.044 (2012). 23178123 PMC3534965

[b30] Shen P. S. *et al.* Protein synthesis. Rqc2p and 60S ribosomal subunits mediate mRNA-independent elongation of nascent chains. Science 347, 75–78, doi: 10.1126/science.1259724 (2015). 25554787 PMC4451101

[b31] Shao S., Brown A., Santhanam B. & Hegde R. S. Structure and assembly pathway of the ribosome quality control complex. Mol Cell 57, 433–444, doi: 10.1016/j.molcel.2014.12.015 (2015). 25578875 PMC4321881

[b32] Shao S. & Hegde R. S. Reconstitution of a minimal ribosome-associated ubiquitination pathway with purified factors. Mol Cell 55, 880–890, doi: 10.1016/j.molcel.2014.07.006 (2014). 25132172 PMC4175178

[b33] Halbach F., Reichelt P., Rode M. & Conti E. The yeast ski complex: crystal structure and RNA channeling to the exosome complex. Cell 154, 814–826, doi: 10.1016/j.cell.2013.07.017 (2013). 23953113

[b34] Matsuda R., Ikeuchi K., Nomura S. & Inada T. Protein quality control systems associated with no-go and nonstop mRNA surveillance in yeast. Genes Cells 19, 1–12, doi: 10.1111/gtc.12106 (2014). 24261871

[b35] Thompson M. K., Rojas-Duran M. F., Gangaramani P. & Gilbert W. V. The ribosomal protein Asc1/RACK1 is required for efficient translation of short mRNAs. Elife 5, doi: 10.7554/eLife.11154 (2016). PMC4848094 27117520

[b36] Majzoub K. *et al.* RACK1 controls IRES-mediated translation of viruses. Cell 159, 1086–1095, doi: 10.1016/j.cell.2014.10.041 (2014). 25416947 PMC4243054

[b37] Inada T. & Aiba H. Translation of aberrant mRNAs lacking a termination codon or with a shortened 3′-UTR is repressed after initiation in yeast. EMBO J 24, 1584–1595, doi: 10.1038/sj.emboj.7600636 (2005). 15933721 PMC1142571

