# Supplementary material for: Ribosome-associated Asc1/RACK1 is required for endonucleolytic cleavage induced by stalled ribosome at the 3′ end of nonstop mRNA
Source: Sci Rep. 2016 Jun 17;6:28234. doi: 10.1038/srep28234 (PMC4911565; doi:10.1038/srep28234)
Supplement: Supplementary Information [file srep28234-s1.pdf]

Supplementary Information for

Ribosome-associated Asc1/RACK1 is required for endonucleolytic cleavage induced by stalled ribosome at the 3' end of nonstop mRNA

Ken Ikeuchi and Toshifumi Inada*

From the Graduate School of Pharmaceutical Science, Tohoku University, Aoba-ku, Sendai 980-8578, Japan

This PDF file includes:

Supplementary Table 1

Table 1 Yeast strains and plasmids used in this study

Strain/Plasmid name	Genotype/Plasmid	Sourse
Strains		
W303-1a	*MAT*a *ade2 his3 leu2 trp1 ura3 can1*	Lab. Stock, Parent
YIT2011	*asc1*Δ::kanMX4	Kuroha *et al.* 2010
YIT2012*	*ski2*Δ::hygMX4, *asc1*Δ::kanMX4	Kuroha *et al.* 2010
YIT2013	*ski2*Δ::kanMX4	Kuroha *et al.* 2010
YIT2014	*dom34*Δ::hygMX4	Kuroha *et al.* 2010
YIT2016	*dom34*Δ::hygMX4, *ski2*Δ::kanMX4	Kuroha *et al.* 2010
YIT2019	*xrn1*Δ::kanMX4	Tsuboi *et al.* 2010
YIT2020	*dom34*Δ::hygMX4, *xrn1*Δ::kanMX4	Tsuboi *et al.* 2012
YIT2035	*asc1*Δ::kanMX4, *dom34*Δ::hygMX4	This study
YIT2036	*hbs1*Δ::natMX4, *dom34*Δ::hygMX4, *ski2*Δ::kanMX4	This study
YIT2037	*asc1*Δ::kanMX4, *dom34*Δ::hygMX4, *ski2*Δ::natMX4	This study
YIT2038	*xrn1*Δ::kanMX4, *asc1*Δ::natMX4	This study
YIT2039	*dom34*Δ::hygMX4, *xrn1*Δ::kanMX4, *hbs1*Δ::natMX4	This study
YIT2040	*dom34*Δ::hygMX4, *xrn1*Δ::kanMX4, *asc1*Δ::natMX4	This study
Plasmids		
p416*GPDp*	*CEN, URA3*	Mumberg *et al.* 1995
p415*GPDp*	*CEN, LEU2*	Mumberg *et al.* 1995
p414*GPDp*	*CEN, TRP1*	Mumberg *et al.* 1995
p413*GPDp*	*CEN, HIS3*	Mumberg *et al.* 1995
p416*GAL1p*	*CEN, URA3*	Mumberg *et al.* 1994
pSA144	*CEN, URA3, GAL1*p*-GFP-FLAG-HIS3*	Dimitrova *et al.* 2009
pIT2004	*CEN, URA3, GPD*p*-GFP-R12-FLAG-HIS3*	Dimitrova *et al.* 2009
pIT2015	*CEN, URA3, GAL1*p*-GFP-Rz-FLAG-HIS3*	Tsuboi *et al.* 2012
pIT2108	*CEN, URA3, GPD*p*-GFP-Rz-FLAG-HIS3*	Kobayashi *et al.* 2010
pIT2156	*CEN, HIS3, GAL1*p*-GFP-Rz-FLAG-HIS3*	This study
pIT2157	*CEN, LEU2, GPD*p*-FLAG-ASC1*	This study
pIT2158	*CEN, LEU2, GPD*p*-FLAG-ASC1 16HNG18AAA*	This study
pIT2159	*CEN, LEU2, GPD*p*-FLAG-ASC1 38RDK40AAA*	This study
pIT2160	*CEN, LEU2, GPD*p*-FLAG-ASC1 65SHI67AAA*	This study
pIT2161	*CEN, LEU2, GPD*p*-FLAG-ASC1 85WDK87AAA*	This study
pIT2162	*CEN, LEU2, GPD*p*-FLAG-ASC1 106HKS108AAA*	This study
pIT2163	*CEN, LEU2, GPD*p*-FLAG-ASC1 127RDK129AAA*	This study
pIT2164	*CEN, LEU2, GPD*p*-FLAG-ASC1 148NDW150AAA*	This study
pIT2165	*CEN, LEU2, GPD*p*-FLAG-ASC1 195HNS197AAA*	This study
pIT2166	*CEN, LEU2, GPD*p*-FLAG-ASC1 216KDG218AAA*	This study
pIT2167	*CEN, LEU2, GPD*p*-FLAG-ASC1 236AQD238AAA*	This study
pIT2168	*CEN, LEU2, GPD*p*-FLAG-ASC1 257ATG259AAA*	This study
pIT2169	*CEN, LEU2, GPD*p*-FLAG-ASC1 175DKM177AAA*	This study
pIT2170	*CEN, LEU2, GPD*p*-FLAG-ASC1 D109Y*	This study
pIT2171	*CEN, URA3, GPDp-ProteinA-TEV-GFP-Rz-HIS3*	This study
pIT2172	*CEN, LEU2, GPDp-RPS2-FLAG*	This study

* The YIT number of these strains has been re-assigned.
